# Dense Neuron Clustering Explains Connectivity Statistics in Cortical Microcircuits

**DOI:** 10.1371/journal.pone.0094292

**Published:** 2014-04-14

**Authors:** Vladimir V. Klinshov, Jun-nosuke Teramae, Vladimir I. Nekorkin, Tomoki Fukai

**Affiliations:** 1 Nonlinear Dynamics Department, Institute of Applied Physics of the Russian Academy of Sciences, Nizhny Novgorod, Russia; 2 Laboratory for Neural Circuit Theory, RIKEN Brain Science Institute, Wako, Saitama, Japan; 3 Laboratory for Nonlinear Oscillatory-Wave Physics, University of Nizhni Novgorod, Nizhni Novgorod, Russia; 4 Department of Bioinformatic Engineering, Osaka University, Suita, Osaka, Japan; 5 PRESTO, Japan Science and Technology Agency, Kawaguchi, Saitama, Japan; 6 Department of Oscillations Theory and Automatic Control, University of Nizhni Novgorod, Nizhni Novgorod, Russia; 7 CREST, Japan Science and Technology Agency, Kawaguchi, Saitama, Japan; University of Pittsburgh, United States of America

## Abstract

Local cortical circuits appear highly non-random, but the underlying connectivity rule remains elusive. Here, we analyze experimental data observed in layer 5 of rat neocortex and suggest a model for connectivity from which emerge essential observed non-random features of both wiring and weighting. These features include lognormal distributions of synaptic connection strength, anatomical clustering, and strong correlations between clustering and connection strength. Our model predicts that cortical microcircuits contain large groups of densely connected neurons which we call clusters. We show that such a cluster contains about one fifth of all excitatory neurons of a circuit which are very densely connected with stronger than average synapses. We demonstrate that such clustering plays an important role in the network dynamics, namely, it creates bistable neural spiking in small cortical circuits. Furthermore, introducing local clustering in large-scale networks leads to the emergence of various patterns of persistent local activity in an ongoing network activity. Thus, our results may bridge a gap between anatomical structure and persistent activity observed during working memory and other cognitive processes.

## Introduction

The organization of neuronal wiring determines the flow of information in neural circuits and hence has significant implications for their function. Cortical neurons often appear to be sparsely connected with a low probability of synaptic connection [1]–[7], suggesting random organization [8]. However, several recent studies reveal nonrandom features of neuronal wiring in cortical circuits including a non-Gaussian distribution of synaptic weights, and non-random patterns of neuronal wiring [2], [9]–[15].

The first feature observed is a long heavy tail in the distribution of excitatory postsynaptic potentials (EPSPs) between cortical neurons [10], [13], [16]. This implies that a small number of very strong connections in local cortical circuits carry a large proportion of the total synaptic weight on a neuron, while the majority of synapses are weak [6], [17]. Such long-tailed (typically lognormal) EPSP distributions of AMPA receptor containing synapses generate spontaneous reverberating activity optimal for spike-based communications by stochastic resonance [18].

The second feature is the highly nonrandom structure of synaptic connections between cortical neurons. The statistics of cortical circuit connectivity have been shown to strongly differ from that of random networks on both local and global scales. In particular, there is evidence that neurons chosen randomly from local cortical circuits exhibit certain connection patterns, or motifs, significantly more often than expected by chance [10], [15], [19]. The third nontrivial feature is correlation between the connection probabilities and synaptic weights. In short, stronger connections are more likely to be found between neurons belonging to certain network motifs [10], [15].

Nonrandom features of synaptic connections create additional complexity in the structure and dynamics of neural networks [12], [16], [18], [20]–[32]. However, the precise connectivity structure of cortical circuits remains elusive. Here, we derive a computational model of network connectivity from the known non-random features of cortical circuits. Our model predicts the typical size of a cluster and defines the statistical relationship between the wiring and weighting.

We then show the significance of such clustering for cortical dynamics both in a small network with a single cluster and a large-scale network with many clusters. In these networks, the clustering of connections is crucial for generating bistable states of neurons belonging to a cluster, which in turn create a rich repertoire of dynamical behavior useful for various types of memory storage.

## Results

Our study is primarily focused on local neuronal circuits of about hundred micrometers in size residing in rat visual cortex. The connection probability among adjacent neurons does not depend strongly on the distance between neurons in the above spatial range, so in our approach we do not consider positions of the neurons. The simplest and most commonly used model for sparsely connected networks is the so-called “random network” (RN) model [33], in which the probability of connections between two arbitrarily chosen neurons is a single constant that doesn’t depend on neuron identity or its location. However, experimental observations suggest that a local cortical network on the scale of about hundred micrometers already demonstrates strong evidence of clustering [10], [15]. Therefore we extended the random network approach to construct a network with clusters (hereafter, we call it “NC”) in which synaptic connections distribute heterogeneously across the network and form a certain number of clustered neuron groups. The rest of neurons in NC do not belong to any cluster. This results in a statistically significant deviation of NC from a random network with the same mean connection probability.

### Sets of Parameter Values Consistent with Experimental Data

**Figure 1:**
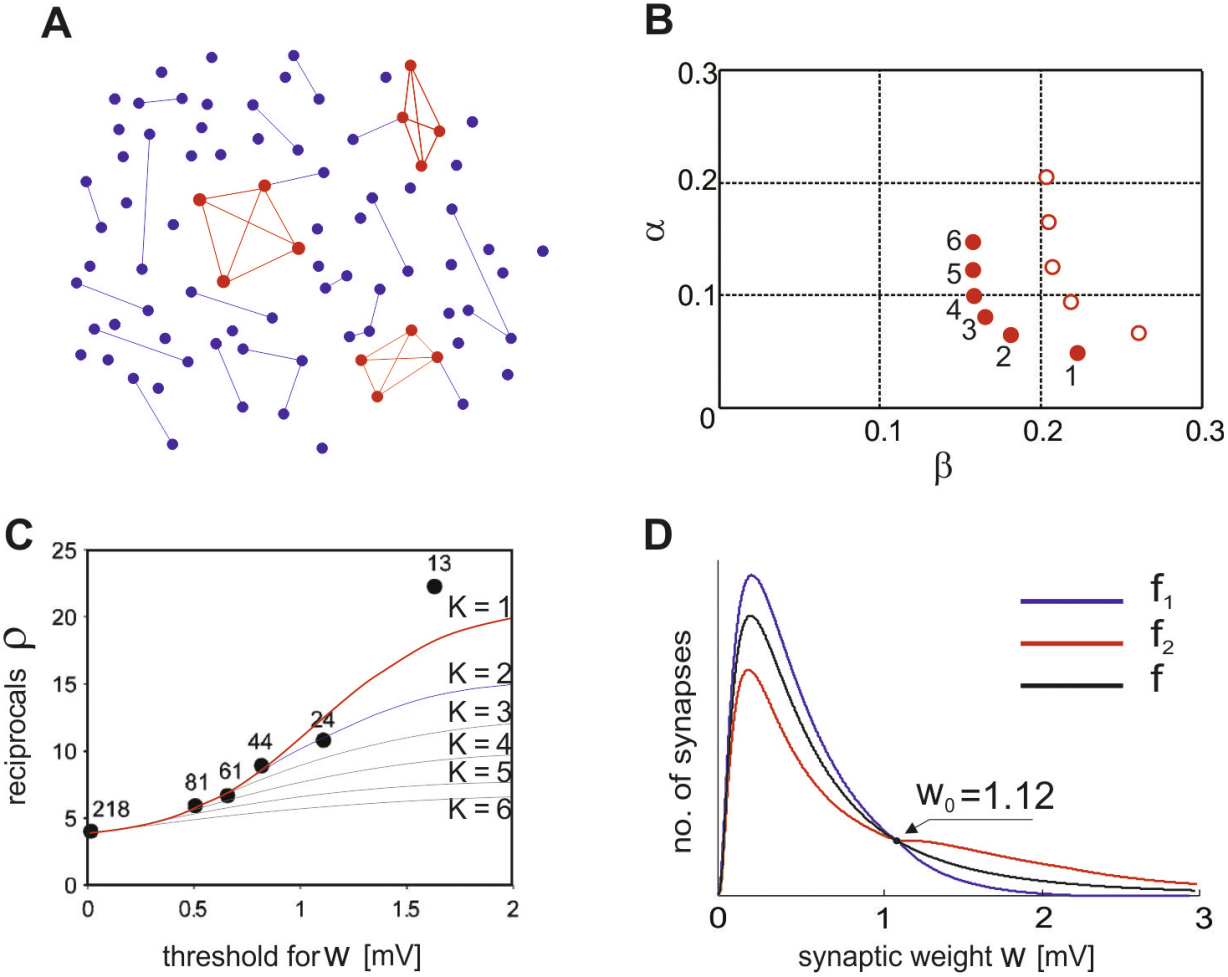
Structure and parameters of the model. **A**, Our NC model is schematically illustrated. The connection probability is higher in clusters (*c*_2_) than outside (*c*_1_). Connections inside and outside clusters are shown in red and blue, respectively. **B**, The parameter sets predicted by the model are depicted on the plane of *α* and *β* with filled circles. The numbers near the circles correspond to the values of *K*. Empty circles depict the sets obtained under the assumption of 10% connections loss in slice preparations. **C**, The experimentally observed threshold-dependence of the overrepresentation of reciprocal connections is fitted by models for different values of *K*. The best fit with *K* = 1 is red, the first after best fit with *K* = 2 is blue, the other fits are black. The number near each data point shows the number of the corresponding connections obtained by experiment. **D**, The probability densities of synaptic weights are shown for *K* = 1: *f*_2_(*w*) stands for connections inside the cluster and *f*_1_(*w*) for all the other connections, and *f*(*w*) for all connections in the entire network. The two distributions have identical values, i.e., *f*_1_(*w*_0_) = *f*_2_(*w*_0_), at the threshold value of *w*_0_ = 1.12 [mV].

Our model is defined as a network of *N* neurons that contains *K* clusters with *M* neurons per cluster (Fig. 1A). Note that *KM* can be smaller than *N*, i.e. some neurons of the network may not belong to any of the clusters. The probability of two units being connected equals *c*_2_ if they belong to the same cluster and *c*_1_ otherwise (if they belong to different clusters, or one of them or both do not belong to any cluster). The statistical properties of a NC are fully determined by the constants *c*_1_ and *c*_2_, the number of the clusters *K* and the relative size of a cluster *β* = *M/N*. It is also convenient to define the coefficient *α* = *Kβ* ^2^, which is the probability that two randomly selected units are both taken from any one of the *K* clusters. All other characteristics of the network may be calculated through these parameters. For example, one can calculate the overall connection probability *c*, the overrepresentation coefficient *R* of reciprocal connections, or the overrepresentation coefficient *T* of triangle motifs (see Materials and Methods).

We examined whether the model can describe experimentally observed nonrandom properties of the connectivity of cortical networks. We first adjusted the values of parameters in the NC model according to the experimental data of local circuits’ connectivity in layer 5 of rat visual cortex [10], and then tested the resultant model on other data from layer 5 of rat somatosensory cortex [15]. From the data reported in the former study one can calculate that the average probability of finding a synaptic connection between a neuron pair was *c* = 0.1157, the coefficient of the overrepresentation of reciprocal connections was *R* = 4.025, and the coefficient of the overrepresentation of triangles was *T* = 2.73.

By analyzing the statistical properties of synaptic connections in the NC model, we obtained value sets of model parameters that agree with the experimental observations (Materials and Methods). We found *six* possible sets of parameter values that meet all the criteria set by experiment. These sets correspond to different numbers of clusters, *K* = 1, 2, … 6 (Fig. 1B, filled circles; see also Fig. S2). We found that no other sets of parameter values for statistical properties consistent with experimental data. The origin of several sufficient parameter sets can be understood as follows. Fixing of the values of *c*, *R* and *T* give *three* conditions for *four* system parameters *c*_1_, *c*_2_, *α* and *β*, which defines a line in the 4D parameter space. The condition that *K* = *α/β* ^2^ is an integer specifies a discrete set of points on this line, while the inequality *Kβ*<1 allows just a finite number (namely six) of these points. The latter inequality implies that the total number of neurons belonging to the clusters should not exceed the total number of neurons in the entire network.

Our model of clustered connections is based on *in vitro* data recorded from slice preparations, in which some fraction *ε* (<1) of synaptic connections could be severed. We simulated whether this loss of connections significantly affects the above results. For this purpose, we estimated the changes in the circuit connectivity induced by the loss of connections and derived the actual values of network parameters for given value of *ε* (Materials and Methods). For example, when *ε* = 10%, we found *five* possible sets of parameters corresponding to *K* = 1, 2, …, 5 (Fig. 1B, empty circles). As a further analysis will show later, the most important parameter set is the one corresponding to *K* = 1, which does not change much after taking into account the connections loss. Thus, we may conclude that the loss of connections in the order of 10% would not significantly change the present results.

### Long-tailed Distributions of Synaptic Weights

Next, we considered what constitutes a likely distribution of synaptic weights within the network. The EPSP distribution can be described with a connectivity matrix *W*, in which element *W_ij_* remains zero in the absence of a connection from neuron *i* to neuron *j*, while in the presence of this connection *W_ij_* (>0) coincides with its weight. In reality, a neuron pair has multiple synaptic contacts and the amplitude of EPSP represents the sum of the contributions from the multiple synapses. The value of *W_ij_* should be interpreted as this sum in our model. Several experiments have shown that the amplitude distribution of EPSPs in cortical circuits is not Gaussian, but closer to a lognormal distribution with a long tail, where the mean and variance were determined to be *µ* = 0.702 [mV] and *σ* = 0.9355 [mV], respectively [10]. Similar long-tailed distributions are known in mouse somatosensory cortex [13] and rat hippocampus [16]. Furthermore, experiments reported that the synaptic weights are positively correlated within certain motifs such as reciprocally connected pairs and neuronal triangles. In general, stronger connections are more often clustered than weaker ones. The experimental evidence for this feature comes from the observation that the overrepresentations of these highly connected motifs increase with an increase in the threshold value [10], [15].

To represent the experimentally observed features, we assume that the distributions of synaptic weights are different between the inside and outside the clusters, where “inside” means a connection linking two neurons belonging to the same cluster, and “outside” means any other connection. Specifically, the two distributions of nonzero synaptic weights were described by different cumulative distribution functions *F*_1,2_(*w*) = Pr (*W_ij_*<*w*) of the EPSP amplitude *w*, where indices 1 and 2 refer to the outside and inside of the clusters, respectively. The weight distributions *f*_1,2_(*w*) are given as the derivatives of the cumulative functions with respect to *w*. Given these functions one can calculate the overrepresentation *ρ*(*w*) of reciprocal connections for connections greater than the threshold *w*. Conversely, by using experimental data for *ρ*(*w*), we can explicitly determine the functions *F*_1_(*w*) and *F*_2_(*w*) that replicate the threshold-dependent statistics of reciprocal connections observed in experiments (see Materials and Methods for the detailed mathematical procedure).

Figure 1C shows the experimentally obtained data for *ρ*(*w*) [10] and the curves fitted by NC for the six sets of parameters obtained earlier. Note that the maximal value of the overrepresentation of reciprocal connections in our model depends on *α* as *ρ*_max_ = 1/*α*. The values of *α* are different among the six parameter sets, and this explains why different levels of *ρ* are reachable for different *K*. The maximum experimentally observed value is *ρ*_max_≈22. Among the six sets of parameter values found earlier, the set corresponding to *K* = 1 gives the best fit with *ρ*_max_≈20.3, while all others yield *ρ*_max_<15. Therefore, our model optimally accounts for all the experimental observations with the set of parameter values *K* = 1, *β* = 0.222, *c*_1_ = 0.07 and *c*_2_ = 1, and this set is employed for further analyses below. The corresponding probability density functions are plotted in Fig. 1D, which shows that strong connections are more represented inside the cluster, while weaker ones are more likely to be found outside. The threshold between “weak” and “strong” connections may be defined as *w*_0_≈1.12 [mV], at which the two probability densities *f*_1_ and *f*_2_ give the same value. These density functions were constructed such that the total probability density *f*(*w*) coincides with a lognormal distribution (Materials and Methods).

However, we should keep in mind that these parameter values were derived from a limited amount of experimental data. In particular, a single data point for *w*>1.5 [mV] in Fig. 1C was crucial for choosing the present value of *K* and more data points are necessary for confirming this choice. The parameter set with *K* = 2 is suitable as well for the replication of the observed dependency *ρ*(*w*) for all data points except the last one. This parameter set describes a network with two clusters, which are a little smaller than the cluster obtained for *K* = 1 (*β* = 0.18) and their membership neurons are interconnected densely but not all-to-all (*c*_2_ = 0.88). Anyway, our connectivity model predicts that local microcircuits in the neocortex of the rat contain clusters comprising approximately one fifth of excitatory neurons. The neurons in the cluster are very densely connected to each other, while the rest of the connections in the network are sparse.

### Excitatory Connection Matrix

**Figure 2:**
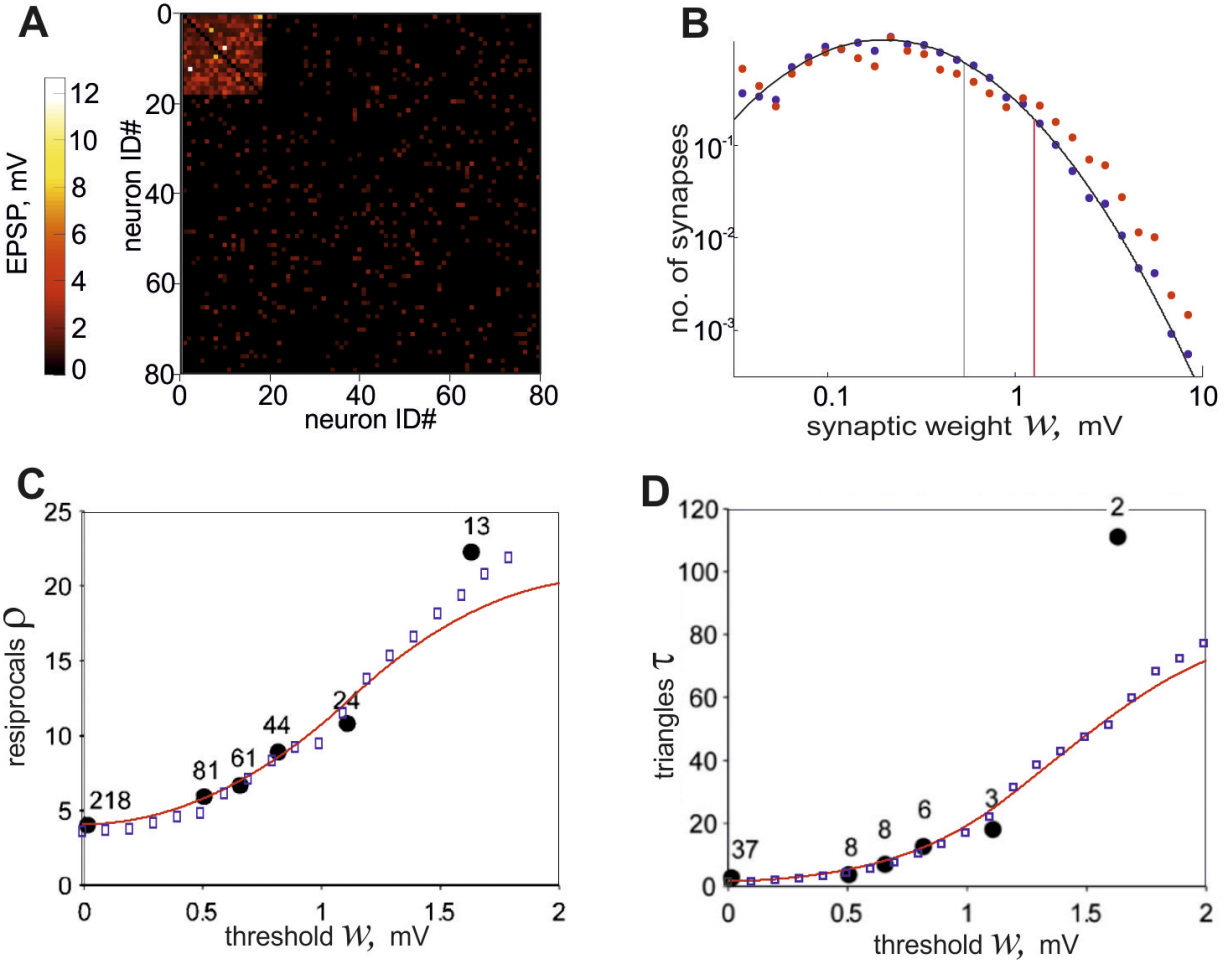
The properties of the network generated by the developed algorithm. **A**, An example of connectivity matrix for 80 excitatory neurons containing a single cluster is shown. The weight of each connection is presented in pseudo color. **B**, The probability density of nonzero weights distribution is shown in logarithmic scales. Black circles show the weight distribution obtained for the entire network and red circles show the distribution inside the cluster. Solid curve represents a lognormal fit to the data points, black vertical line corresponds to the mean weight of connections for the entire network and red line corresponds to the mean weight inside the cluster. **C**, The coefficients of the overrepresentation of reciprocal connections *ρ* versus threshold value *w* are shown. Blue squares are obtained in the model network and black circles are taken from the experimental data shown in ref. [10], where each number shows the number of the corresponding connections obtained by experiment. Red line is calculated by formula (7) for the parameter choice of *K* = 1. **D**, The coefficients of the overrepresentation of triangles *τ*(*w*) are shown. Symbols are used in the same manner as in the previous plot.

We numerically generated connection matrices for excitatory neurons according to our model one of which is represented in Fig. 2A. Since our model describes small local cortical domains, our network consisted of *N* = 80 excitatory neurons. This number was chosen partly because the minimal functional module of neocortex, the so-called minicolumn, is considered to comprise 80 to 100 neurons [34]–[37]. This number also roughly corresponds to the number of neurons in a box with 100 micrometer side of the neocortex. Since the density of neurons is 3∼6×10^5^/mm^3^ in the rat cortex, such a cortical volume contains about 300∼600 neurons [3], [38]–[43]. However, another estimate gives a smaller number of about 50 [44].

To visualize the small-scale network, we arranged the 80 neurons in such a way that the first (*M* = *βN* = ) 18 of them belonged to the cluster. The connections within the cluster are dense and strong, while all the rest of the connections are sparse and weak. Figure 2B presents the distribution of nonzero synaptic weights in the entire network. Black dots stand for the overall weight distribution of the network, which is close to a lognormal fit 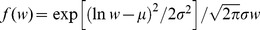, with the mean *µ* = 0.702 and variance *σ* = 0.9355. Red dots represent the distribution of synaptic weights within the cluster, which clarifies that stronger connections are more often found among them. As indicated by two vertical lines, the mean weight of the connections is larger inside the cluster as well.

The generated connection matrix involves overrepresented two-neuron and three-neuron network motifs. The overrepresentation coefficients obtained numerically in the network are plotted for various thresholds in Fig. 2C for reciprocal connections and in Fig. 2D for triangle motifs. The experimental data [10] and the theoretical curves are also plotted in the same axis for comparison. The characteristics of the small network generated by our model were in good agreement with those of the experimental data. This coincidence is not surprising for the reciprocal connections because we calculated the weight distributions of our model based on the data. However, the quantitative agreement for triangle motifs was unexpected because we did not make use of the statistical data for triangles in adjusting parameter values of the model. It is important that the observed overrepresentation of triangles is not just the reflection of the overrepresentation of reciprocal connections (see Materials and Methods), so this agreement between the model and experimental data supports the plausibility of our model.

### Consistency of the Connectivity Model with Other Experimental Data

**Figure 3:**
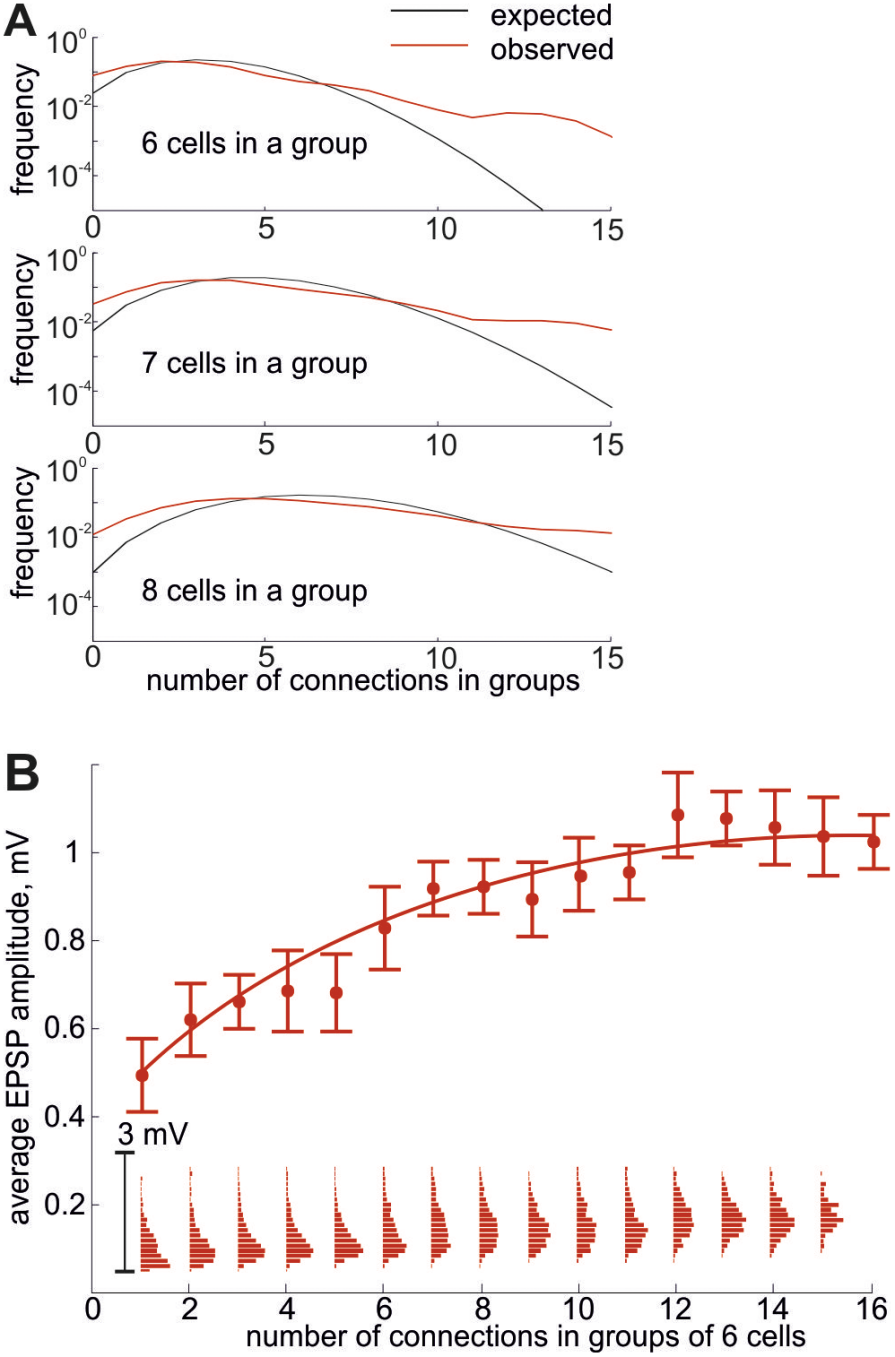
Statistics of connections in groups of neurons in the model. **A**, The expected (black) and observed (red) frequencies of neuron groups are plotted for given number of connections. Only groups of 6, 7 and 8 neurons are considered. **B**, The mean EPSP amplitude in a group of 6 neurons is plotted as a function of the number of connections in the group. Insets show the distributions of the EPSP amplitude in the corresponding groups.

Our model of synaptic connectivity was derived mainly through statistical data obtained from the layer 5 of rat visual cortex. However, the presence of clustered neurons or subnetworks has been reported in other layers, areas and species including layer 5 of rat somatosensory cortex [2], [15], layer 2/3 of rat visual cortex [11], and layer 2/3 of mouse barrel cortex [14]. In particular, various network parameters were measured in somatosensory cortex by Perin et al. [15] demonstrating significant overrepresentations of highly connected motifs for larger neuron groups than those reported in ref. [10]. Our model qualitatively replicates these results as shown in Fig. 3A. Supposing a random connectivity in the network, we obtain the binomial distribution of the number of connections (gray curve). Adding of a cluster of densely connected neurons increases the probability to find a group with more connections inside it, uplifting the distribution for large numbers of connections (black curve). Thus, the present clustering well explains the overrepresentations of highly connected groups.

Perin et al. also found an interesting relationship between the number of connections among a group of six neurons and the average synaptic weight for the group: the synaptic weight grows with the number of connections and almost saturates after 20% of possible connections are formed. We examined whether our network model with a cluster is consistent with this saturating property of higher network motifs by carrying out a similar analysis in groups of six neurons that were chosen randomly from our model. As shown in Fig. 3B, the average EPSP amplitude for a group depends on the number of connections among six neurons in the group, and this function saturates in a qualitatively similar fashion to what was described in ref. [15]. Insets show the resultant distributions of the EPSP amplitude in neuron groups with different numbers of connections.

Pajevic and Plenz also analyzed the organization of strong connections in various complex real-world networks including local cortical networks [31]. They found that in the latter ones the clustering coefficient of connections is positively correlated with their weight rank. We also calculated the clustering coefficient of synaptic connections in our model and found that it is positively correlated with the EPSP amplitude with a correlation coefficient ranging between 0.52 and 0.55.

### Bistable Dynamics of the Network with a Cluster

In previous sections we have shown that local cortical circuits are likely to contain relatively large clusters of very densely connected neurons. Next we explored how these features may affect circuit dynamics, for which we compared the activity of model networks with and without the clustering structure. For this sake we considered (i) the NC with the set of parameter values described above and (ii) the RN having the same mean connection probability and EPSP distribution as the NC. Both networks consisted of 80 excitatory and 20 inhibitory neurons. In both networks, the mean connection probability of excitatory neurons *c*≈0.12, and the mean amplitude of nonzero EPSPs *w*≈0.8 [mV]. The NC also contained a cluster of 18 neurons that were fully connected to each other with the mean strength of *w*≈1.1 [mV]. The overall EPSP distribution in both networks was lognormal, which was previously demonstrated to enable spontaneous low rate activity in large-scale networks [18]. However, because the present network is very small, it is not capable of self-maintained activity, thus requiring external input. Therefore we introduced uncorrelated spike inputs to all neurons in the network with identical statistical characteristics (see Materials and Methods for the model details).

**Figure 4:**
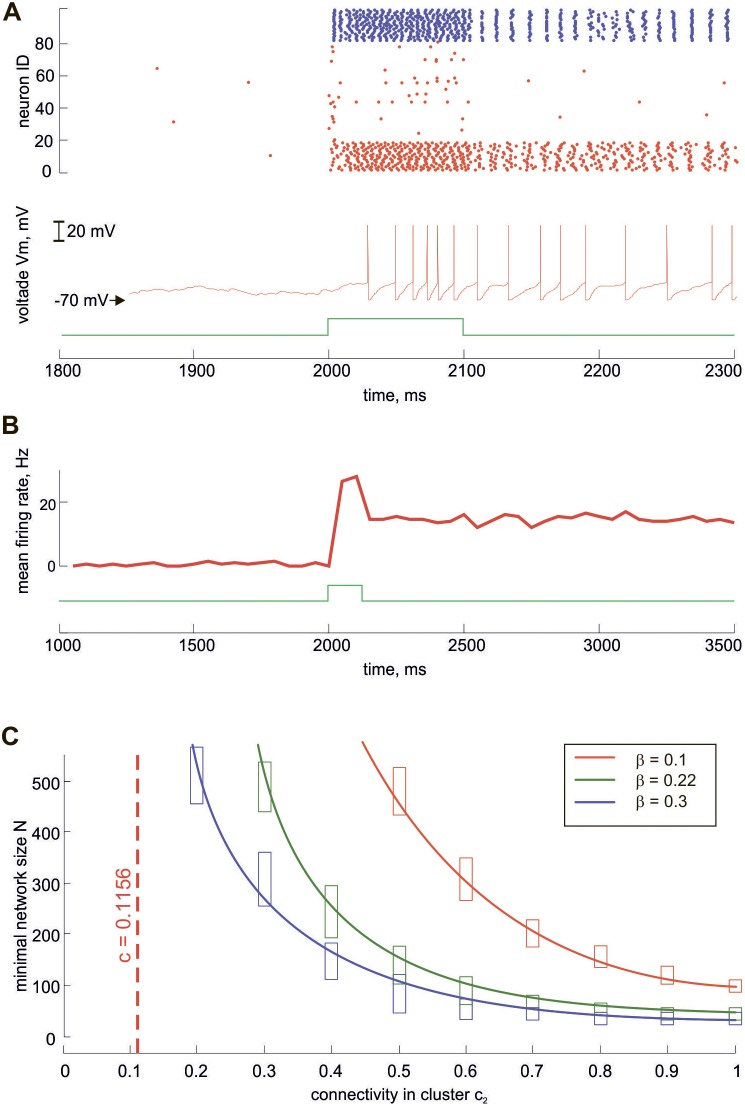
Bistability of the NC dynamics. **A**, The network switching from the “low” to the “high” state by a transient external simulation. Top: spike raster of the network, red dots for excitatory and blue dots for inhibitory neurons. The clustered neurons have identities from 1 to *K* = 18. Bottom: output voltage of one of the clustered neurons (red) and the external signal (green). **B.** Stability of the low and the high states. The network is simulated by a transient pulse lasting 100 ms (green curve). The mean firing rate of the network (red curve) is around zero before the stimulation, then increases and remains elevated (∼20 Hz) even when the stimulation is terminated. Note that A and B use different scales of the time axes. **C.** The minimal network size *N* sufficient for the bistability emergence depending on the connectivity inside the cluster *c*_2_. The curves of different colors correspond to various values of *β* = 0.1 (red), *β* = 0.22 (green), *β* = 0.3 (blue).

When weak external noisy input was applied to the RN, all neurons started to fire irregularly at a low mean frequency of about 1 Hz. We refer to this regime as the “low state”. The dynamics of the NC also shows a low state similar to that of the RN. Interestingly, however, the NC had another regime in which neurons showed firing with a much higher mean frequency of about several tens of spikes per second. We call this state “high state”. The network state is changed from low to high state by a brief additional stimulation (Fig. 4A). In the high state the clustered neurons fire at high rates, while the rest of the network rarely produces spikes. The firing patterns of the clustered and non-clustered neurons are irregular and asynchronous, as indicated by a wide power spectrum without any pronounced peaks (see Fig. S3).

Importantly, in NC both low and high states are stable, as illustrated in Fig. 4B, meaning that the network with a cluster becomes bistable. Neural networks can be bistable without clusters if the nonlinear response of neurons and the strength of coupling are adequately tuned. Therefore, clustering is not a necessary condition for bistability. While the crucial contribution of clusters to bistability will be shown later in large-scale networks, it is worthwhile pointing out that the clustering of connections allows bistability in rather small networks. To show this, we carried out a parametric study of the NC. We varied the relative cluster size *β* and connectivity inside the cluster *c*_2_, keeping the average connectivity (*c* = 0.1156) and average coupling strength (*w* = 0.8) unchanged. For several values of *β*, we changed *c*_2_ from the minimal value *c*_2_ = *c* (no clustering) to the maximal value *c*_2_ = 1 (ultimate clustering) and looked for the minimal network size *N* sufficient for the emergence of bistability (Fig. 4C). For *β* = 0.22 and maximal clustering (*c*_2_ = 1), the high state is stable already for *N* = 40. At a medium level of clustering (*c*_2_ = 0.5) the bistability requires at least *N* = 140 neurons. Without clustering (*c*_2_ = *c*), the bistability emerges only for *N*>1000. We also obtained similar results for other values of the relative size of clusters (*β* = 0.1 and *β* = 0.3). Thus, the clustering of connections dramatically reduces the minimal network size to generate the bistability.

### Various Forms of Persistent Activity in Large-scale Networks with Clusters

As shown above, the clustering of connections makes the activity of a local cortical circuit bistable. However, local circuits in the brain do not function in isolation from the adjacent networks, and the local clustering of synaptic connections may also have nontrivial effects on large-scale network dynamics. This issue was previously tested in large-scale networks of autonomously active spiking neurons [30], and we now tested it in large-scale networks of (non-autonomous) integrate-and-fire neurons with clusters. To this end, we introduced multiple clusters into a network model that was previously constructed to account for asynchronous irregular firing of cortical neurons at low frequencies (the mean frequency of 1–2 Hz) [18]. The network consisted of 10000 excitatory neurons and 2000 inhibitory neurons, and excitatory-to-excitatory connections initially were homogeneous, sparse, random, and obeyed a lognormal EPSP distribution (see Materials and Methods for details of the model). To introduce multiple clusters into the network model, we partitioned it into smaller subnetworks each consisting of *N_s_* neurons and reorganized the connections inside each subnetwork according to the NC model. Therefore, each subnetwork contained *βN_s_* (*β*≈0.22) clustered neurons. The connections between the subnetworks were not changed. As a result, we obtained a network with local clusters of densely connected neurons, sparse connectivity on the global scale and log-normal distribution of EPSPs. The network received no external background input.

**Figure 5:**
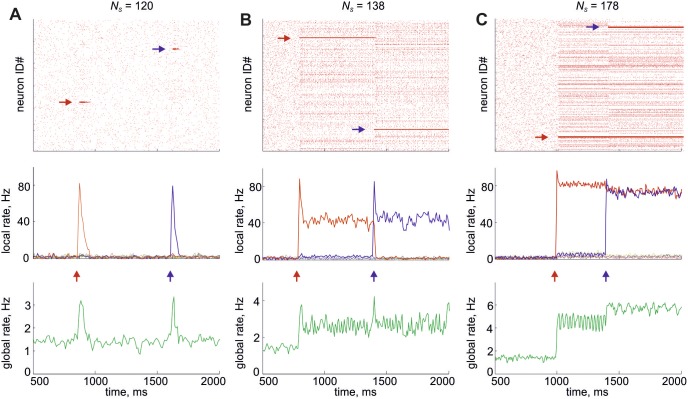
Implications of the local clustering on the large-scale dynamics. **A–C,** The spike rastergrams of all 10000 excitatory neurons (top), the averaged firing rate of the local subnetworks (middle) and the averaged firing rate of the entire network (bottom) are shown for different values of the size of each subnetwork: *N_s_* = 120 (A), 138 (B) and 178 (C). In the middle panels the firing rates of different subnetworks are shown by different colors. Arrows in top and middle panels indicate the clusters of neurons activated by brief external stimuli and the stimulus timing, respectively. In (B) and (C), some neurons outside the active clusters also exhibit slightly higher firing rates if they are projected to by sparse strong synapses from these clusters.

The dynamics of the large-scale network acquires several novel features after the introduction of local clusters. The network preserves ability to generate stable low-rate asynchronous irregular activity without background input. This spontaneous activity is possible because each neuron receives sufficiently strong recurrent input in the large-scale network, unlike in the small network studied above. In addition, the network becomes capable of demonstrating regimes with localized high-rate activity reminiscent of the high state of the small network (Fig. 5). In such a regime, one or more local circuits may be elevated to the high state by brief external stimuli and produce spikes at a high rate while the rest of the network fires sparsely. The mean firing rate of the entire network increases slightly though it remains low.

We performed a series of simulations varying the size of each subnetwork *N_s_*. We found that the stability of the high state of a local subnetwork strongly depends on its size. For relatively small values of *N_s_*, the high state is only metastable: the subnetwork activated by external input fires at higher rates for about 100 ms, but it gradually returns to the low state (Fig. 5A). For larger *N_s_*, the high state is stable: the activated subnetwork settles in the high state and continues to fire at a high rate until another subnetwork is activated by external input. Because of recurrent inhibition in the large-scale network, competition emerges between different subnetworks. For this reason, only one subnetwork may be in the high state for any given moment, and if any subnetwork previously was in the high state it switches back to the low state when the other one is set in the high state (Fig. 5B). Further growth of *N_s_* reduces the competition and allows several subnetworks to be in the high state simultaneously (Fig. 5C).

## Discussion

### The Connectivity Model

We have presented a model of synaptic connectivity in local cortical circuits based on the non-random statistical properties measured previously from small volumes of rat visual cortex. Our model assumes the existence of dedicated subsets of clustered neurons with different connectivity and different distributions of synaptic weights inside and outside the clusters. Our model incorporates several major features of the connectivity of cortical synapses observed experimentally: (i) The probability of finding highly-connected small groups of neurons is higher than expected in random networks; (ii) Synaptic connections are on average stronger between members of those highly-connected groups; (iii) The overall distribution of EPSPs obeys a log-normal distribution with a long tail. The fact that all of these statistical properties are replicated by our model demonstrates its biological plausibility.

The present model of the network with clusters is based on *in vitro* data recorded from slice preparations, in which a portion of connections could be severed. Long-range connections are more likely to be lost, but some local ones may too be severed. We have simulated the effects of this cut in our model and found that it does not significantly change the present results if the percentage of the cut is in the order of 10%. This is because the subgroup of very densely connected neurons remains densely connected even after the cut of some connections.

Other models of clustered connections between cortical neurons have been reported previously. A clustered network of fifty layer 5 pyramidal neurons was statistically reconstructed based on experimental data [10]. An algorithm was introduced to construct a network model from a random network using the distance-dependent connection probabilities measured in experiment [15]. The most distinctive feature of the present model is that, unlikely with the previous ones, we explicitly consider the interplay between the distributions of synaptic weights and the clustering of synaptic connections. To this end, the EPSP distributions in our model are different inside and outside of the cluster. This heterogeneity explains the threshold-dependent statistics of highly-connected motifs (Fig. 2) and the saturating growth of the mean EPSP amplitude versus the number of connections within certain motifs (Fig. 3). It is also noteworthy that though our model primarily concerns layer 5 of the rat visual cortex, the model was shown to be consistent with recent experimental observations coming from other cortical areas [15], [31]. These results suggest that the features of nonrandom connectivity and clustering that we have explored may be applicable more widely across different brain areas.

Our model makes several predictions about the wiring architecture of local cortical circuits, which can be in principle experimentally tested. The first prediction is that small volumes of neocortex contain relatively large clusters of excitatory neurons that are very densely connected with one another. Neurons in a cluster comprise up to about one fifth of the total cortical population. Thus, local subnetworks consisting of about one hundred neurons contain clusters of about twenty densely interconnected neurons. Secondly, the amplitude distributions of EPSPs are predicted to have different profiles in neurons belonging to a cluster and in those not belonging to it (Fig. 1D). Though the experimental confirmation of a full connectivity diagram of cortical neurons seems to be difficult at present, computer-automated reconstruction techniques in combination with modern image processing technologies, such as connectomics [46]–[49], may identify such a circuit diagram in the near future.

It is also intriguing to study a relationship between the clustering predicted by our model and the function-specific anatomical connections in local cortical circuits. A number of recent studies report that the connection probability between cortical neurons depends on their functional properties. For example, the layer 2/3 of the primary somatosensory cortex displays a subset of highly-active pyramidal neurons that are likely to be connected to each other more often than others [14]. The connectivity between layer 2/3 neurons in visual cortex depends significantly on the similarity of visually evoked neuronal responses [64] and the pattern of inter-layer projections within local cortical circuits [11]. To clarify how neural clusters are represented in the function-specific connectivity is an important open problem.

### Implications for the Network Dynamics

We have shown that an immediate consequence of the proposed synaptic clustering is the generation of a bistable regime in the network dynamics with distinct “high” and “low” states. To explain why clustering leads to the emergence of the stable high state let us consider RN and NC consisting of *N* = 80 excitatory neurons studied above. Both NC and RN models have the same mean connection probability equal approximately to 12% and mean EPSP amplitude equal approximately to 0.8 mV. In a network of 80 neurons, this means that on average 10 neurons project to each neuron with the total EPSP amplitude of approximately 8 mV. The excitation threshold is about 20 mV, so this EPSP size is not enough to excite a postsynaptic neuron in a RN even if all presynaptic neurons are excited. Thus, neurons are activated mostly by external input to RN, which corresponds to the low state.

In contrast, in the case of NC there exists a subset of 18 neurons forming a densely connected cluster. In this cluster all neurons are interconnected with relatively large mean EPSP amplitudes of approximately 1.1 mV. Therefore, each of them receives the total EPSP amplitude of approximately 19 mV from other clustered neurons, which is almost enough to elicit action potentials from postsynaptic neurons belonging to the cluster. The lack of input can be covered by input from the rest of the network and from external signal, such that once the cluster is activated, the clustered neurons continue to fire persistently and the network remains in a high activity state.

One may achieve bistability in the recurrent network without clusters just by strengthening the connections or increasing the network size [52], [55]. Our parametric study shows that networks without clustering demonstrate bistability only when they become very large (*N*∼1000), while with clustering they become bistable for much lower values of *N*∼100. This also holds true for different cluster sizes (*β* = 0.1 and *β* = 0.3 are tested). Therefore, if the predictions of our model about the cluster size and connectivity were inaccurate, the main conclusions would remain correct: relatively large and dense clusters lead to the emergence of bistability in small-scale cortical circuits.

Bistable behavior is considered to play an important role for various neural computation tasks [52], such as temporal integration in decision making and interval timing [53], [54] and working memory [55]–[58]. Here, we have demonstrated this phenomenon in a network with a biologically relevant connectivity structure, and provided a computational estimate for the minimal size of cortical circuits that would be bistable. It is noteworthy that this size roughly corresponds to the number of cells in a “minicolumn” [2], [36], which may be a minimal functional module of neocortex. We also note that our model demonstrates persistent firing states in sensory cortex, the area in which a number of recent experiments have suggested the presence of working memory, including V4, MT, inferotemporal cortex (IT), primary and secondary somatosensory cortices [59], [60]. Persistent firing states were also previously demonstrated in auditory cortex [61], [62] and a cultured neural network of the hippocampus [63], for which a long-tailed EPSP distribution was recently discovered [16].

While the bistable behavior of a small network clearly shows a possible role of clustered connections, the role of sparse non-clustered connections remains unclear in the small network. We have revealed a possible role of non-clustered connections in the dynamics of large-scale networks. In such a network, sparse non-clustered connections generate a low-frequency spontaneous activity, which was previously shown to be optimal for spike-based communication [18]. On the background of this low-rate activity, clustered connections create local spots displaying high-rate activity. If the clusters innately exist in neocortex, our results may support the Lego theory of memory [50], although whether they can self-organize by synaptic plasticity through sensory experiences remains to be an interesting question.

The stability of this elevated activity and the number of clusters that can be co-activated simultaneously were shown to depend on the size of the clusters. There may be other ways to achieve the simultaneous activation of multiple clusters in a large-scale network. For instance, in a rate-based competitive network model the number of winner neural units surviving in the competition is determined by the balance between self-inhibition on each unit and lateral inhibition between units [51]. In short, increasing self-inhibition weakens relative effects of lateral inhibition and hence enables more units to survive. Therefore, if some inhibitory neurons only inhibit those excitatory neurons belonging to the same cluster (this introduces effective self-inhibition in the subnetwork), more clusters may be co-activated in the entire network. However, changing the size of each cluster yields a simple, yet useful method to control the number of simultaneously available memory modules in local cortical circuits without the kind of fine-tuning such as achieving a specific connectivity pattern.

The influence of clustering of connections on large-scale network dynamics was previously studied by Litwin-Kumar and Doiron [30]. They showed that clustering of connections induces slow dynamics in the network, producing transient increases or decreases of the firing rate in clusters of neurons. Thus, these high and low activity states appear to be metastable in their model, while in our network they are stable and switched on or off by external stimuli. A crucial difference between the two models is that neurons in their network are in a suprathreshold regime and fire autonomously even without any input. In contrast to this, neurons in our model are in a subthreshold regime, and the low-rate spontaneous firing emerges from the collective network dynamics generated by sparse non-clustered connections.

In the conclusion we should note that though we proposed a simple rule to embed subnetworks with clusters in a large-scale network, a biologically plausible model of connections in large cortical volumes remains to be constructed based on experimental data. In particular, in such attempts, it is necessary to take into account the observed distance-dependent effects on connectivity [6] and clustering [15]. Construction of realistic models of large-scale networks and understanding the potential role of connectivity statistics in neural computations are important open questions that deserve further study.

## Materials and Methods

### The Overrepresentation of Reciprocal Connections

In the NC of *N* neurons with *K* clusters each consisting of *M* neurons and (*N*-*KM*) neurons belonging to none of the clusters, the mean connectivity, i.e., the probability that two randomly chosen units are connected, is given as
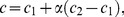
(1)where *α* = *KM* ^2^/*N* ^2^. The presence of clusters results in strong differences in the statistical properties of synaptic connections between the NC and the random network (RN) with the same probability of connection *c*. Particularly, it results in the growth of the number of highly connected motifs in the network. Thus, the probability of two units to be reciprocally connected in RN equals *P_R_* ^0^ = *c* ^2^, whereas in the NC this probability is equal to *P_R_* = *c*_1_^2^+*α* (*c*_2_^2^–*c*_1_^2^). This difference in the probabilities leads to the following coefficient characterizing the overrepresentation of reciprocal connections in the NC with regard to the RN:
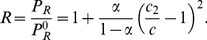
(2)


### The Overrepresentation of Triangle Motifs

The probability of finding a “triangle” consisting of three neurons all interconnected to each other (reciprocally or unidirectionally) in the NC is made up of the probabilities of the following three cases, with *p_i_* being the probability of finding a neuron triplet of the described type and 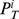 being the probability that they make a triangle.

Case 1. None of three neurons belong to the same cluster:
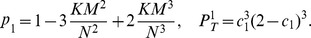



Case 2. Two neurons belong to the same cluster:
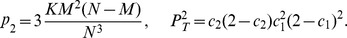



Case 3. All three neurons belong to the same cluster:
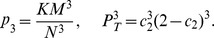



All these probabilities were calculated approximately for *N, M* >>1. To calculate the total probability of finding a triangle one must take into account all three types of triplets as
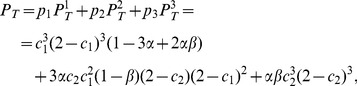
(3)where the coefficients *α* = *KM* ^2^/*N* ^2^ and *β* = *M*/N.

This probability calculated for NC is larger than the probability *P_T_* ^0^ = *c* ^3^ (2–*c*)^3^ of finding a triangle in RN. It is important that the overrepresentation of high-order motifs is not just the reflection of the overrepresentation of popular lower-order patterns. Given the overrepresentation coefficient *R* of reciprocal connections, we can calculate the probability of finding a triangle as *P_T_* ^1^ = *c* ^3^ (2–*Rc*)^3^ from Equation 2, which is still lower than *P_T_*. We define the ratio *T = P_T_/P_T_* ^1^ as the coefficient of the overrepresentation of triangle motifs.

### Sets of Parameter Values Consistent with the Connectivity of Cortical Circuits

Here we explain the method to obtain the experimentally observed statistical quantities, i.e., *c* = 0.1157, *R* = 4.025 and *T* = 2.73 [10], by adjusting the values of parameters in the NC. The tunable parameters in the model are the connection probabilities *c*_1_ and *c*_2_, the number of clusters *K* and the coefficient *α*. The last two parameters define the relative size of each cluster *β = M*/*N = 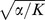
*. First, we examine the range of acceptable values of *α*. Given *c* and *R*, we can express *c*_1_ and *c*_2_ by using (1) and (2) as follows:
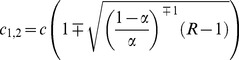
where plus or minus sign is adopted for c_1_ and c_2_, respectively. The obvious conditions 0≤*c*_1,2_≤1 give the range of possible values of *α* as



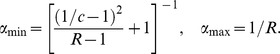
The experimentally observed values of *c* and *R* give *α* ∈ [0.05, 0.25].

In the next step we derive a possible value range for *β.* For this purpose we investigate the coefficient *T* of the overrepresentation of triangles versus *α* for different fixed values of *β* (Fig. S1). The plot of *T*(*α*) shifts upwards as the value of *β* increases, and the target value *T* = 2.73 is obtainable in the narrow interval of *β* ∈ [0.156; 0.222]. Then, given the intervals of *α* and *β,* taking into account that *K* = *α/β* ^2^ must be an integer and *Kβ*<1 must hold, we found *six* possible sets of parameter values that meet all the criteria set by experiment. These sets are depicted in Fig. 1 B (solid circles) and Fig. S2 and correspond to different numbers of clusters, *K* = 1, 2, …, 6. The cluster size was different for each of the sets and varied within the range *β* ∈ [0.16; 0.22].

### Simulations of Possible Connection Cuts in Slice Preparation

It is expected that slice recordings sever some fraction of connections between neurons, which obviously influences our estimates of the circuit connectivity. In order to take into account the effect of the possible connection cut, we investigated how the estimated values of model parameters may change if we suppose that a certain fraction *ε* (<1) of connections is randomly cut before the measurements. In this case, the actual count of connections before the cut should be (1−*ε*)^−1^ times greater than it was measured, and the actual number of motifs with *X* connections should be (1−*ε*)^−*X*^ times greater. Using these expressions and the previously derived equations, we can calculate the actual values of *c*, *R* and *T* from the observed values for any value of *ε*. These values correspond to the “reconstructed” circuit before the connection loss. For example, when *ε* = 10% we obtain *c* = 0.129, *R* = 4.05 and *T* = 3.36, and these values were used to obtain the *five* possible sets of values for *c*_1_, *c*_2_, *α* and *β*, each corresponding to *K* = 1, 2, …, 5 with different values of *β* ∈ [0.2, 0.26]. The five parameter sets are shown in Fig. 1D (empty circles).

### The Weight Distributions

We suppose that the distribution of nonzero synaptic weights inside and outside neuron clusters is given by two different cumulative distribution functions *F*_1,2_(*w*) = Pr(*W_ij_*<*w*), where index 1 or 2 refers to the outside and inside, respectively. Then the cumulative distribution of synaptic weights in the overall network equals
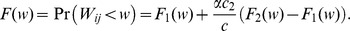
From this formula, we can calculate the overrepresentation *ρ* of reciprocal connections for synaptic connections greater than the threshold *w* as
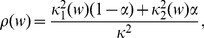
(4)where *κ*_1_, *κ*_2_ and *κ* are the probabilities to find a connection (> *w*) outside the clusters, inside the clusters and in the overall network, respectively. These probabilities can be found from the weights distributions as *κ*_1,2_(*w*) = c_1,2_(1−*F*_1,2_(*w*)) and *κ* = *κ*_1_(1−α)+*κ*_2_α. Since *F*_1,2_(0)  = 0, taking zero threshold *w* = 0 gives *ρ*(0)  = R.

If the functions *F*(*w*) and *ρ*(*w*) are determined from experimental data, we can explicitly derive the expressions of the cumulative weight distributions as
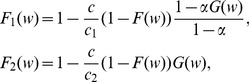
(5)where



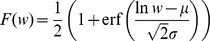
is the cumulative distribution function for the lognormal distribution 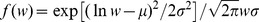. In the above expression, erf(*x*) is the error function and the function



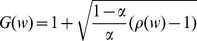
depends only on the overrepresentation function *ρ*(*w*). Thus, choosing the cumulative functions *F*_1,2_(w) according to Equation 5, we can obtain a desired function *ρ*(*w*) in the network. We note that the maximal value of *ρ* depends on *α* and is given as *ρ*_max_ = 1/*α.* This property is important for fitting experimental data with our model.

### Simulation of Local Circuit Dynamics

To simulate a local cortical circuit we used a network model based on the model from [55]. This model describes neural networks quite realistically and includes AMPA, NMDA and GABA synapses, spike-frequency adaptation and short-term depression. The network model consists of two populations on *N_E_* excitatory and *N_I_* = 0.25*N_E_* inhibitory neurons. Each excitatory neuron is described by the following equation:
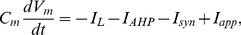
(6)

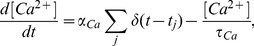
(7)Here, *C*_m_ = 0.5 nF is the membrane capacitance, *V*_m_ is the membrane voltage, *I_L_* = *g_L_*(*V_m_*–*V_L_*) is the leak current, *g_L_* = 0.025 µS, *V_L_* = –70 mV, *I_AHP_* = *g_AHP_*[*Ca* ^2+^](*V_m_* –*V_K_*) is a calcium-activated potassium current for spike-frequency adaptation, *I_syn_ = I_AMPA_+I_NMDA_+I_GABA_* is the recurrent synaptic input from the rest of the network. If *V_m_* reaches the threshold *V*_thr_ it is reset to *V_L_* and is held there for the refractory period *τ_r_ = *2 ms. The inhibitory neurons are described by (6) with *I_AHP_* = 0 (no spike-frequency adaptation) and *g_L_* = 0.05 µS.

The synaptic currents are given by the equations *I_AMPA_* = *g_AMPA_s_AMPA_*(*V_m_*–*V_E_*), *I_NMDA_* = *g_NMDA_s_NMDA_*(*V_m_ –V_E_*)/(1+[*Mg* ^2+^]exp(−0.062*V_m_*)/3.57), *I_GABA_* = *g_GABA_s_GABA_*(*V_m_*–*V_I_*), where *V_E_* = 0 mV, *V_I_* = –70 mV, *g_AMPA_* = 2.3 µS, *g_NMDA_* = 0.16 µS, *g_GABA_* = 0.1 µS, [*Mg^2^* ^+^] = 1 mM. The gating variables *s* for each type of receptors are described by the second-order kinetics.
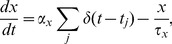
(8)

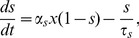
(9)where the sum runs over presynaptic spike times. For the AMPA channels *τ_x_* = 0.05 ms and *τ*_2_ = 2 ms, for the NMDA channels *τ_x_* = 2 ms and *τ_s_* = 80 ms, and for the GABA receptors *τ_x_* = 0.1 ms and *τ_s_* = 10 ms. *α_s_* = 1 for all types of the receptors, and the values of *α_x_* were varied to control the size of EPSPs/IPSPs.

The connectivity and EPSPs between the excitatory neurons were chosen according to the connectivity model developed earlier. The relation between the parameters *α_x_* for the AMPA and NMDA synapses were chosen so that the impacts of the AMPA and NMDA currents in the EPSPs were approximately equal (varying the ratio between these currents led to the change of the phase response curves of the neurons and the degree of their synchronization [45]). The excitatory-to-inhibitory, inhibitory-to-excitatory and inhibitory-to-inhibitory connections were set all-to-all with *α_x_* randomly distributed in the interval [0;0.1]. The synaptic delays were randomly distributed from 1 to 3 ms for all types of the connections. The external input *I_app_* was a Poisson train on AMPA-like pulses with *α_x_* = 0.1 and intensity *λ* = 100 Hz.

### Simulation of Large-scale Networks Dynamics

When we studied the dynamics of the large-scale network with clusters, we used a simpler model based on the one from [18]. The network consists of 10000 excitatory and 2000 inhibitory neurons, with sparse random coupling and probabilities of 10% for excitatory and 50% for inhibitory connections. The neurons were described by the leaky integrate-and-fire model with conductance-based synaptic currents:
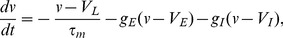
(10)where *v* is the membrane potential, *τ_m_* is the membrane decay time constant, *V_L_*, *V_E_*, and *V_I_* are the reversal potentials of leak current, excitatory and inhibitory postsynaptic currents, respectively. Excitatory and inhibitory synaptic conductance, *g_E_* and *g_I_*, is described by the following equation:
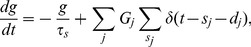
(11)where τs is the decay constant of synaptic current. The second term represents synaptic inputs from presynaptic neurons, Gj and dj are the weight and delay of synaptic input from the j-th presynaptic neuron, and sj is the spike timing of the neuron. Values of the parameters were set as VL = –70 mV, VE = 0 mV, VI = –80 mV, τm = 20 ms for excitatory neurons and 10 ms for inhibitory neurons and τs = 2 ms. Spike threshold is Vthr = –50 mV and the refractory period is 1 ms.

Initially the values of *G_ij_* for excitatory-to-excitatory synapses are selected such that the corresponding EPSPs are distributed according to the lognormal distribution. Then, to introduce clustering all the excitatory neurons were partitioned into groups of *N_S_* units each, and the connections inside each group were rewired according to the present NC model. The values of *G_ij_* for the other types of connections were selected as of *G_ij_* = 0.018, 0.002 and 0.0025 for excitatory-to-inhibitory, inhibitory-to-excitatory and inhibitory-to-inhibitory synapses. Synaptic delays were chosen randomly from the uniform distribution [*d*_0_−1, *d*_0_+1] with mean *d*_0_ set as 2 ms for excitatory-to-excitatory connections and 1 ms for the other types of connections. Synaptic transmission fails between excitatory neurons according to the following EPSP-dependent rate *p* = *a*/(*a*+*EPSP*), where *a* = 0.1 mV.

## Supporting Information

Figure S1 **The plot of T versus α for different values of β.** Blue horizontal line shows the target experimental value T = 2.73, red lines correspond to boundary values of β for which the target value is obtainable.
(TIF)

Figure S2 **Possible parameter sets in the model.** The values of parameters α, β, c1 and c2 are plotted for six parameter sets corresponding to K = 1, 2, … 6. All these parameter sets give the experimentally measured values of the overall connectivity c = 0.1157, the overrepresentation of reciprocal connections R = 4.025 and that of triangle motifs T = 2.73.
(TIF)

Figure S3 **Bistable dynamics of the network.** (A) Mean field of the network (blue) and the external input (green). (B) Power spectrum in the low state. (C) Power spectrum in the high state.
(TIF)
