# Supplementary figures and images for: Dense Neuron Clustering Explains Connectivity Statistics in Cortical Microcircuits
Source: PLoS One. 2014 Apr 14;9(4):e94292. doi: 10.1371/journal.pone.0094292 (PMC3986068; doi:10.1371/journal.pone.0094292)

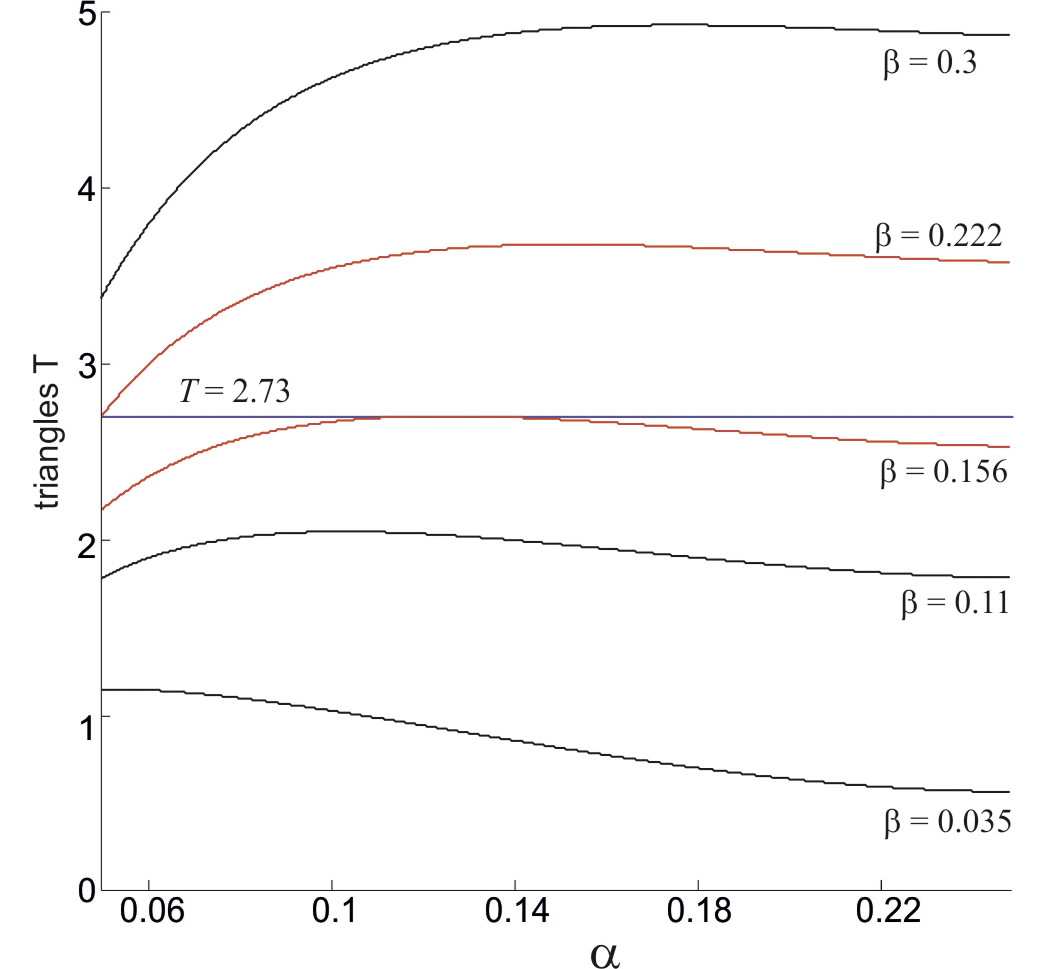

Supplement: Figure S1 — The plot of T versus α for different values of β. Blue horizontal line shows the target experimental value T = 2.73, red lines correspond to boundary values of β for which the target value is obtainable. (TIF) [file pone.0094292.s001.tif]

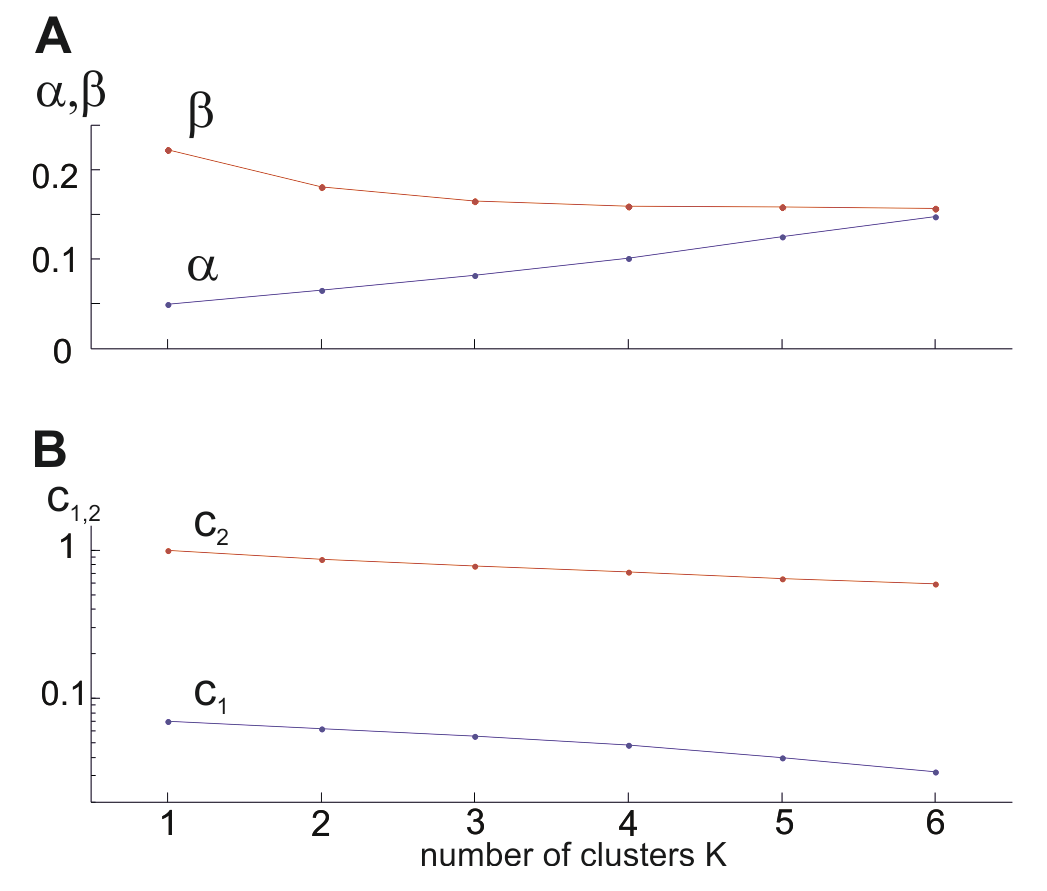

Supplement: Figure S2 — Possible parameter sets in the model. The values of parameters α, β, c1 and c2 are plotted for six parameter sets corresponding to K = 1, 2, … 6. All these parameter sets give the experimentally measured values of the overall connectivity c = 0.1157, the overrepresentation of reciprocal connections R = 4.025 and that of triangle motifs T = 2.73. (TIF) [file pone.0094292.s002.tif]

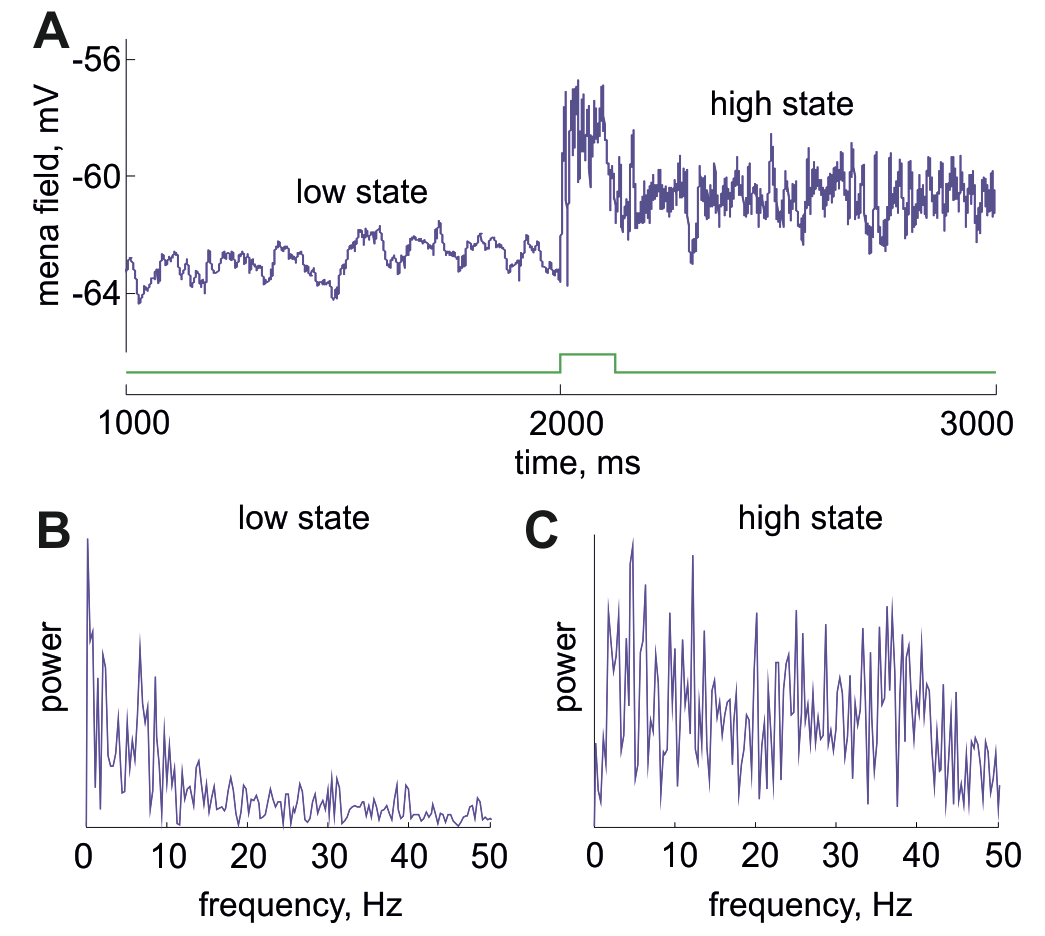

Supplement: Figure S3 — Bistable dynamics of the network. (A) Mean field of the network (blue) and the external input (green). (B) Power spectrum in the low state. (C) Power spectrum in the high state. (TIF) [file pone.0094292.s003.tif]
